# Assessment of the cardiac output at rest and during exercise stress using real-time cardiovascular magnetic resonance imaging in HFpEF-patients

**DOI:** 10.1007/s10554-024-03054-6

**Published:** 2024-01-18

**Authors:** Alexander Schulz, Hannah Mittelmeier, Lukas Wagenhofer, Sören J. Backhaus, Torben Lange, Ruben Evertz, Shelby Kutty, Johannes T. Kowallick, Gerd Hasenfuß, Andreas Schuster

**Affiliations:** 1 Department of Cardiology and Pneumology, University Medical Center Göttingen, Georg-August University, Robert-Koch-Str. 40, 37099 Göttingen, Germany; 2 German Center for Cardiovascular Research (DZHK), Partner Site Göttingen, Göttingen, Germany; 3 Institute of Biomedical Imaging, University of Technology Graz, Graz, Austria; 4 Department of Cardiology, Campus Kerckhoff of the Justus-Liebig-University Giessen, Kerckhoff-Clinic, Bad Nauheim, Germany; 5 Taussig Heart Center, Johns Hopkins Hospital and School of Medicine, Baltimore, MD 21287 USA; 6 Institute for Diagnostic and Interventional Radiology, University Medical Center Göttingen University Medical Center Göttingen, Georg-August University, Göttingen, Germany; 7 School of Biomedical Engineering and Imaging Sciences, King’s College London, London, UK

**Keywords:** Exercise stress CMR, HFpEF, Real-time imaging, Cardiac output

## Abstract

This methodological study aimed to validate the cardiac output (CO) measured by exercise-stress real-time phase-contrast cardiovascular magnetic resonance imaging (CMR) in patients with heart failure and preserved ejection fraction (HFpEF). 68 patients with dyspnea on exertion (NYHA ≥ II) and echocardiographic signs of diastolic dysfunction underwent rest and exercise stress right heart catheterization (RHC) and CMR within 24 h. Patients were diagnosed as overt HFpEF (pulmonary capillary wedge pressure (PCWP) ≥ 15mmHg at rest), masked HFpEF (PCWP ≥ 25mmHg during exercise stress but < 15mmHg at rest) and non-cardiac dyspnea. CO was calculated using RHC as the reference standard, and in CMR by the volumetric stroke volume, conventional phase-contrast and rest and stress real-time phase-contrast imaging. At rest, the CMR based CO showed good agreement with RHC with an ICC of 0.772 for conventional phase-contrast, and 0.872 for real-time phase-contrast measurements. During exercise stress, the agreement of real-time CMR and RHC was good with an ICC of 0.805. Real-time measurements underestimated the CO at rest (Bias:0.71 L/min) and during exercise stress (Bias:1.4 L/min). Patients with overt HFpEF had a significantly lower cardiac index compared to patients with masked HFpEF and with non-cardiac dyspnea during exercise stress, but not at rest. Real-time phase-contrast CO can be assessed with good agreement with the invasive reference standard at rest and during exercise stress. While moderate underestimation of the CO needs to be considered with non-invasive testing, the CO using real-time CMR provides useful clinical information and could help to avoid unnecessary invasive procedures in HFpEF patients.

**Supplementary Information:**

The online version contains supplementary material available at 10.1007/s10554-024-03054-6.

## Introduction

The cardiac output (CO) is known as an important parameter for the estimation of the global cardiac function and is being used as a cardiac performance index to diagnose, monitor and prognosticate patients with cardiovascular disease including heart failure or pulmonary hypertension [1, 2, 3, 4].

For the calculation of the CO different methods have been described and validated including thermodilution during right heart catheterization (RHC), echocardiography, partial carbon dioxide rebreathing, or cardiovascular magnetic resonance imaging (CMR) [5, 6, 7].

While thermodilution still is considered as one of the most accurate methos for the calculation of CO in clinical practice [7, 8], CMR offers the chance for robust non-invasive image acquisition, independent of the patient characteristics or acquisition-angle.

First validation studies have shown good agreement of the CO as calculated by RHC with the CO as calculated by CMR by different methods including volumetric calculations or conventional phase-contrast imaging [7].

While those methods mostly allow for an accurate calculation at rest, some diseases require the assessment of the cardiac functional reserve during physiological exercise stress, as affected patients compensate cardiac failure at rest. This applies for example to patients with heart failure with preserved ejection fraction (HFpEF) [9, 10]. Real-time CMR potentially allows reliable quantification of cardiac function during exercise stress in HFpEF but has not been validated against an invasive reference standard yet [11, 12]. Therefore this methodological study aimed to introduce and validate a novel approach applying real-time phase-contrast imaging and directly compare it to RHC for the quantification of the CO at rest and during exercise stress in HFpEF patients.

## Methods

### Patient cohort and study design

Within the HFpEF-Stress trial 75 patients with dyspnea on exertion (NYHA ≥ II), preserved ejection fraction (EF > 50%) and signs of diastolic dysfunction (E/e’ ≥ 8) were prospectively recruited for further evaluation [9]. After exclusion due to unexpected findings in CMR, 68 patients were included in the final study cohort [9].

All patients underwent rest and stress RHC followed by rest and exercise stress CMR within 24 h [9]. Patients had to be in stable sinus rhythm during the examinations.

The collected data was used for a retrospective calculation and comparison of the CO as measured during CMR and RHC.

The study was approved by the local ethics committee, and all patients gave written informed consent prior to participation. The study was conducted according to the principles of the Helsinki Declaration and was funded by the German Center for Cardiovascular Research (DZHK-17, Clinicaltrials.gov: NCT03260621).

### Right heart catheterization cardiovascular magnetic resonance exercise stress protocol

#### Right heart catheterization

Details of the protocol and procedure of RHC have been published previously [9, 13]. Patients were characterized according to RHC measurements of the pulmonary capillary wedge pressure (PCWP) as masked HFpEF, only diagnosed during exercise stress with a PCWP ≥ 25mmHg or overt HFpEF, diagnosed at rest already with a PCWP ≥ 15mmHg. The remaining patients served as a control group with non-cardiac dyspnea.

#### Cardiac magnetic resonance imaging

CMR was performed on a 3.0T Magnetom Skyra (Siemens Healthcare, Erlangen, Germany) using a 32-channel cardiac surface receiver coil. Details of the protocol have been described [9]. At rest, volumetric assessments of the left ventricle (LV) were obtained from balanced steady state free precession sequences in short-axis positioning with full coverage of the atria and the ventricles (temporal resolution: 30 frames per cardiac cycle, time to echo (TE) 1.5ms, time of repetition (TR) 55ms, flip angle 55°, 7 mm slice thickness with 7.7 mm inter-slice gap).

In addition, a 2D phase-contrast gradient-echo sequence with retrospective electrocardiographic gating was acquired at rest (temporal resolution: 30 frames per cardiac cycle, flip angle 20°, TE/TR 2.5/37, in-plane resolution: 1.8 mm x 1.8 mm x 6 mm). Appropriate velocity encoding was selected beforehand, and scans were repeated if aliasing occurred. Real-time phase-contrast acquisitions were obtained of the ascending aorta in the same slice position at rest and during exercise with a temporal resolution of 44ms at a minimum TE/TR of 3.15/2.46. Further details of the real-time scan parameters have been published earlier [14].

Quantitative flow measurements were obtained from the ascending aorta in a cross-sectional orientation above the aortic valve in the same slice position for both, conventional and real-time phase-contrast imaging. Inter- and intra-observer variability was assessed in ten randomly selected cases by two independent observers.

All CMR images were evaluated using semi-automated analysis in cvi42 (version 5.13, Circle Cardiovascular Imaging, Calgary, Canada).

### Calculation of cardiac output

During RHC, CO at rest and during exercise stress was measured by thermodilution as a mean of three consecutive repeats and indexed to the body surface area as previously described [9]. CO measurements by RHC were considered as the reference standard.

**Fig. 1:**
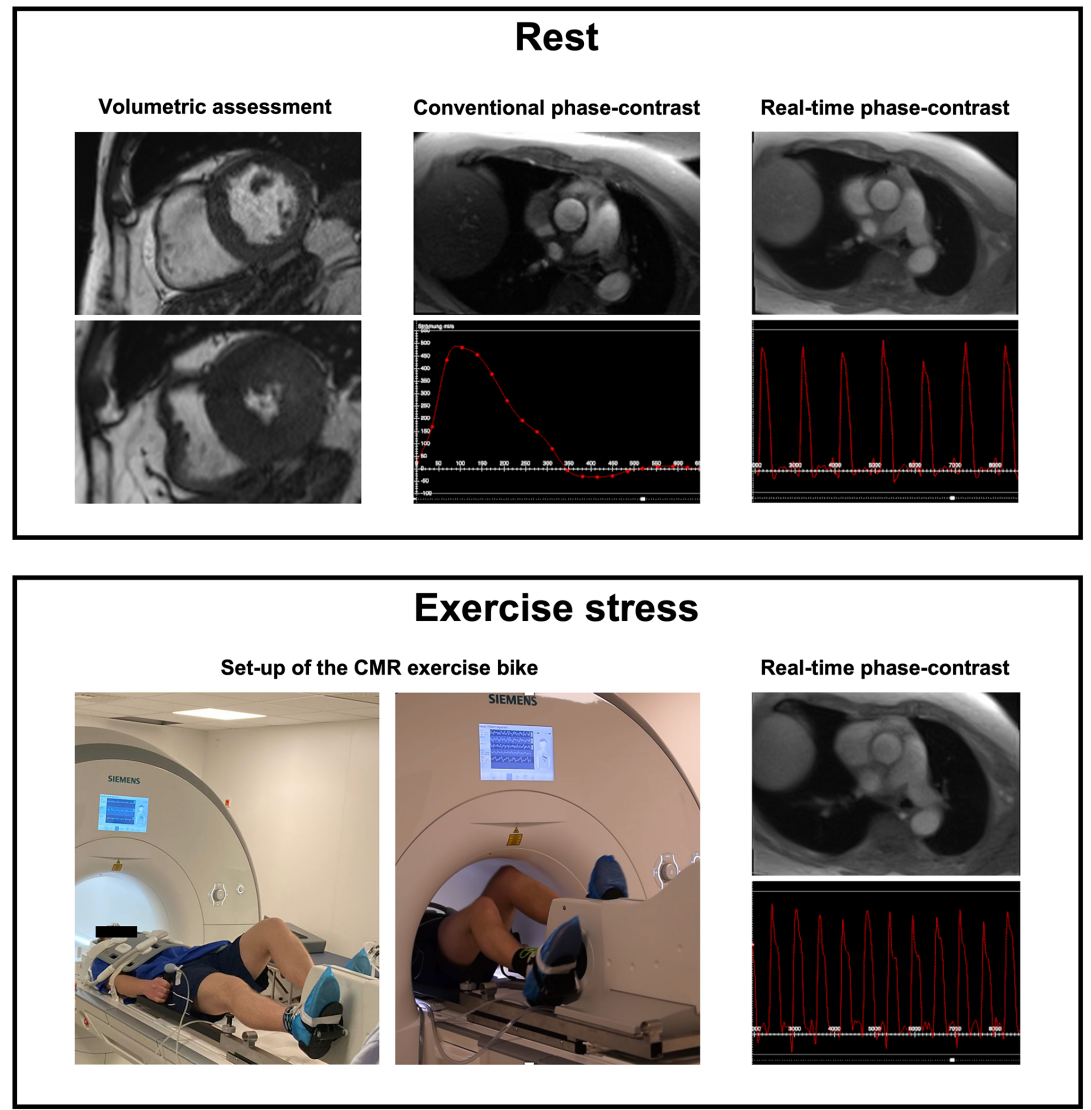
CMR methods to calculate the cardiac output. The top box displays the methods to quantify the cardiac output at rest including volumetric assessments, conventional phase-contrast blood flow measurements and real-time phase-contrast blood flow measurements. The bottom box shows the set-up of the exercise bike in the CMR scanner as well as an example for the quantification of the cardiac output using real-time phase-contrast imaging. CMR – cardiovascular magnetic resonance

Calculation of CO by CMR was performed using three different approaches (see Fig. 1):

1. Volumetric assessment (rest):

CO was calculated using volumetric assessments of the left ventricular (LV) end-diastolic and end-systolic volume. Afterwards, the LV stroke volume (SV) was calculated as the difference of end-diastolic and end-systolic volume and multiplied by the heart rate.

2. Conventional phase-contrast assessments (rest):

In phase-contrast images, the SV was measured as the effective forward blood flow-volume in the ascending aorta during a single heartbeat and multiplied by the heart rate.

3. Real-time phase-contrast assessments (rest and stress):

A dedicated analysis in a custom-built tool for Python (version 3.10.0, Python Software foundation, Delaware, United States) was used to calculate real-time CO. In short, the beat-to-beat stroke volume was assessed and multiplied by the heart rate. Afterwards, the mean of the individual measurements was calculated and generated as final output.

To explore clinical differences in CO between patients with HFpEF and controls, the CO was divided by the body surface area and is presented as the indexed CO.

### Statistics

All statistical analysis were performed using SPSS version 27.0 (IBM, Armonk, NY) and GraphPad Prism 9 (GraphPad Software, California, United States). Normal distribution was tested with a Shapiro-Wilk-test. For continuous variables results are plotted as mean ± standard deviation or median ± inter quartile range, respectively. Categorial variables are reported as frequencies with corresponding percentages. Differences between the individual methods for the calculation of the CO were compared using a Wilcoxon rank test. Differences between HFpEF and control patients were assessed by a Mann-Whitney-U Test. Inter- and intra-observer variability, as well as the agreement of the individual methods was calculated by an intra-class correlation coefficient (ICC) and is reported with the corresponding 95% confidence interval. Results of the ICC were interpreted as suggested by Bobak et al. [15]. Furthermore, the coefficient of variation (CoV) was calculated as CoV = $\frac{\text{s}\text{t}\text{a}\text{n}\text{d}\text{a}\text{r}\text{d}\ \text{d}\text{e}\text{v}\text{i}\text{a}\text{t}\text{i}\text{o}\text{n}\ \text{o}\text{f}\ \text{d}\text{i}\text{f}\text{f}\text{e}\text{r}\text{e}\text{n}\text{c}\text{e}\text{s} }{\text{m}\text{e}\text{a}\text{n}\ \text{o}\text{f}\ \text{d}\text{i}\text{f}\text{f}\text{e}\text{r}\text{e}\text{n}\text{c}\text{e}\text{s}}$. In addition, Bland-Altman plots were used to visualize the mean bias between individual methods and RHC. The limits of agreement of the Bland-Altman plots were verified by testing the correlation of the means of methods with their mean difference.

## Results

### Baseline characteristics

In total, 19 patients were diagnosed with masked HFpEF (only diagnosed during exercise stress) and 15 patients with overt HFpEF (diagnosed at rest already). The remaining 34 patients were assigned to the control group with non-cardiac dyspnea.

Clinical details have been published earlier [9].

No differences were found in heart rates between examinations by RHC and volumetric LV stroke volume assessment at rest (71 vs. 70 bpm; *p* = 0.797). The heart rates during the assessments of conventional and real-time phase-contrast measurements at rest were lower compared to RHC (conventional phase-contrast: 66 vs. 71 bpm; *p* < 0.001, real-time phase-contrast: 62 vs. 71 bpm; *p* = 0.005) (compare Table 1). Comparing individual CMR examinations at rest, patients had higher heart rates during volumetric LV stroke volume assessments than during conventional and real-time phase-contrast measurements, while no difference between the latter two techniques was observed (compare Supplemental Fig. 1). During exercise stress real-time phase-contrast CMR examination heart rates again were slightly lower compared to exercise stress RHC (101.5 vs. 108.0 bpm; *p* = 0.014) (See Table 1).

**Table 1:** Comparison of the cardiac output calculated by right heart catheterization and cardiovascular magnetic resonance imaging

Parameters RHC	Parameters CMR		*p*-value RHC vs. CMR
**HR rest (bpm)**			
71.5 (64.2;77.8)	LV-SV	70.0 (63.0;77.0)	0.797
	PC-CMR	66.0 (58.0;74.0)	< 0.001
	RT-CMR	62.0 (60.0;72.0)	0.005
**CO rest (l/min)**			
5.7 (4.6;6.8)	LV-SV	6.8 (5.2;8.0)	< 0.001
	PC-CMR	5.2 (4.3;6.4)	< 0.001
	RT-CMR	5.0 (4.2;6.0)	< 0.001
**HR stress (bpm)**			
108.0 (100.0;112.5)	RT-CMR	101.5 (96.0;110.0)	0.014
**CO stress (l/min)**			
10.4 (8.1;12.8)	RT-CMR	8.6 (7.5;14.0)	< 0.001

Independent continuous parameters are presented as medians with interquartile ranges. The *p*-value refers to the difference between measurements during RHC and CMR. HR – heart rate, CO – cardiac output, RHC – right heart catheterization, CMR – cardiovascular magnetic resonance imaging, LV-SV – left ventricular volumetric stroke volume, PC – conventional phase-contrast, RT – real-time phase-contrast

### Calculation of the cardiac output

#### Rest measurements

**Fig. 2:**
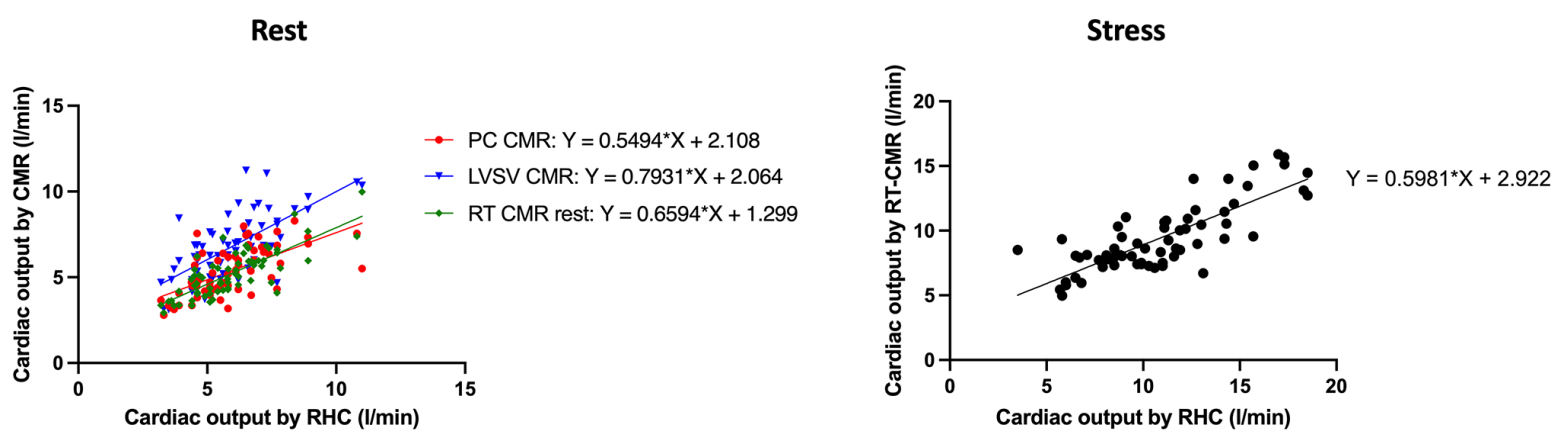
Linear regression of the cardiac output as measured by CMR and RHC. Resting measurements are displayed on the left. The curves are fitted regression lines of the agreement of the cardiac output as measured by RHC with conventional PC CMR (red curve, circles), LV stroke volume (blue, triangles) and RT CMR (green, diamonds). Real-time measurements during exercise stress and their agreement with RHC are shown on the right. CMR – cardiovascular magnetic resonance imaging, RHC – right heart catheterization, PC – phase-contrast, LVSV – left ventricular stroke volume, RT – real-time

**Fig. 3:**
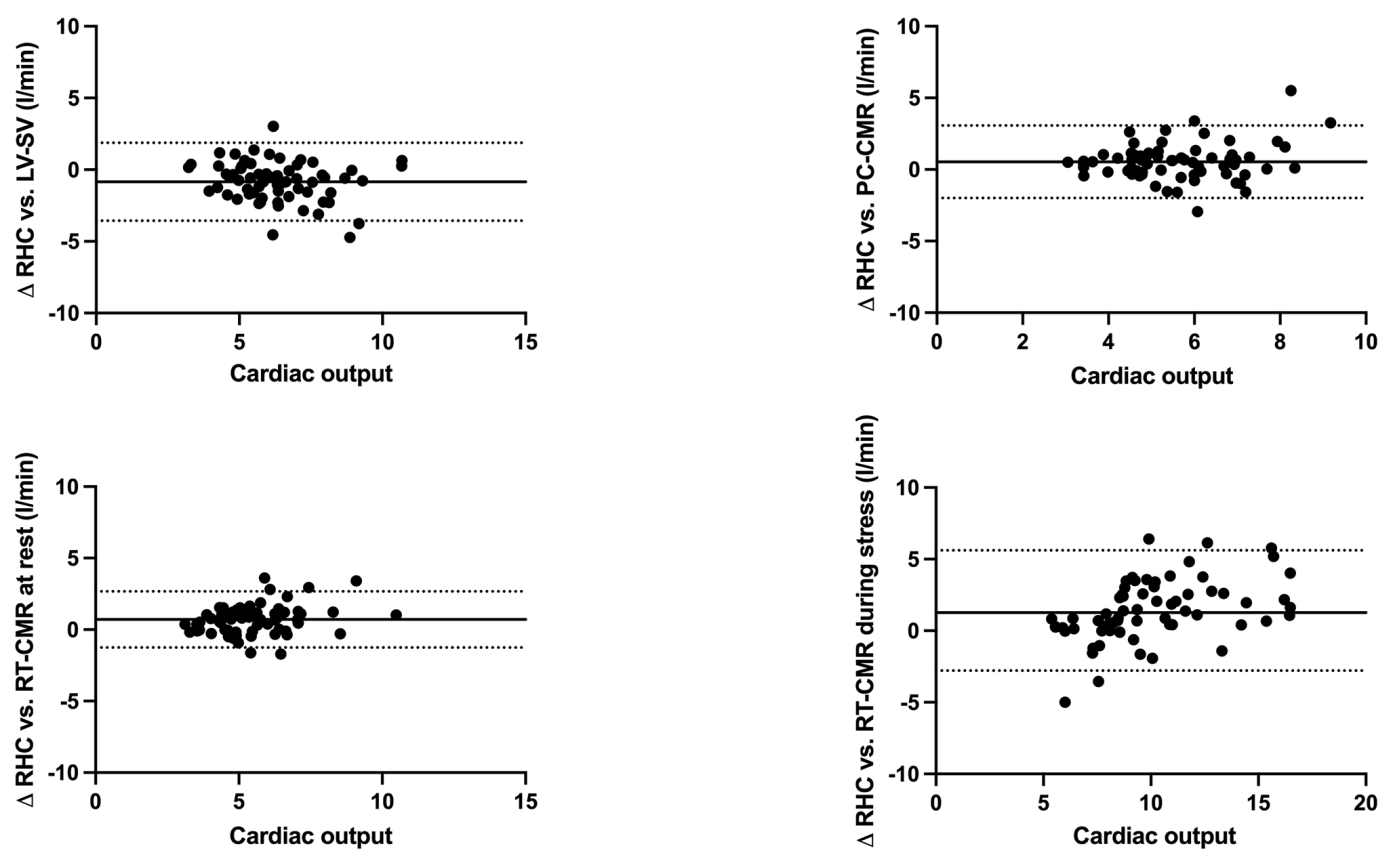
Bland-Altman-Plots of individual methods for the calculation of the cardiac output in CMR compared to the reference standard of RHC. The upper row and the bottom left image show measurements at rest, while the bottom right displays measurements during exercise stress. The continuous line marks the mean bias between two methods. The dotted lines confine the 95% limits of agreement. CMR – cardiovascular magnetic resonance, RHC – right heart catheterization, LV-SV – left ventricular stroke volume, PC –conventional phase-contrast imaging, RT – real-time phase-contrast imaging

At rest, all methods for the calculation of CO showed good agreement with measurements by RHC with an ICC of 0.772 for conventional phase-contrast measurements and an ICC of 0.872 for real-time phase-contrast measurements. Furthermore, real-time phase-contrast measurements had the lowest CoV of 17.1% (see Table 2; Fig. 2). While the CO was overestimated by calculations using the LV stroke volume, conventional and real-time phase-contrast imaging underestimate the CO (see Table 2; Fig. 3). No correlation of the means of methods with their individual differences could be found for rest measurements (LV stroke volume: *r*=-0.21; *p* = 0.089, conventional phase-contrast: *r* = 0.05; *p* = 0.661, real-time phase-contrast: *r* = 0.24; *p* = 0.060).

**Table 2:** Agreement of the cardiac output calculated by different methods in cardiovascular magnetic resonance imaging compared to right heart catheterization

Methods	ICC (95% CI)	*p*-value	Limits of agreement (95% CI)	CoV (%)
RHC vs. LV-SV	0.810 (0.691–0.883)	< 0.001	-0.9 (-3.6–1.9)	22.5
RHC vs. PC-CMR	0.772 (0.631–0.860)	< 0.001	0.5 (-2.0–3.1)	21.1
RHC vs. RT-CMR rest	0.872 (0.791–0.921)	< 0.001	0.7 (-1.3–2.7)	17.1
RHC vs. RT-CMR stress	0.805 (0.611–0.894)	< 0.001	1.4 (-2.8–5.6)	28.5

HR – heart rate, CO – cardiac output, RHC – right heart catheterization, CMR – cardiovascular magnetic resonance imaging, LV-SV – left ventricular volumetric stroke volume, PC – conventional phase-contrast, RT – real-time phase-contrast, ICC – intra-class correlation coefficient, CoV – Coefficient of variation, CI – Confidence interval

#### Stress measurements

During exercise stress, calculations of the CO by real-time phase-contrast imaging had good agreement with measurements by RHC (ICC 0.805; *p* < 0.001) (see Table 2; Fig. 2). However, the CoV was higher compared to CO measurements at rest (CoV stress: 28.5%; CoV rest: 17.1%). Concordantly to the resting measurements, the CO was underestimated by real-time CMR during exercise stress (see Table 2; Fig. 3). When analyzing the Bland-Altman plots, we found a positive correlation of the means of methods with their differences (*r* = 0.53; *p* < 0.001).

Inter- and intra-observer agreement for real-time phase-contrast measurements of the CO was excellent both at rest and during exercise stress with an ICC > 0.985 (compare Supplemental Table S1).

#### Real-time CMR cardiac index in HFpEF patients

**Fig. 4:**
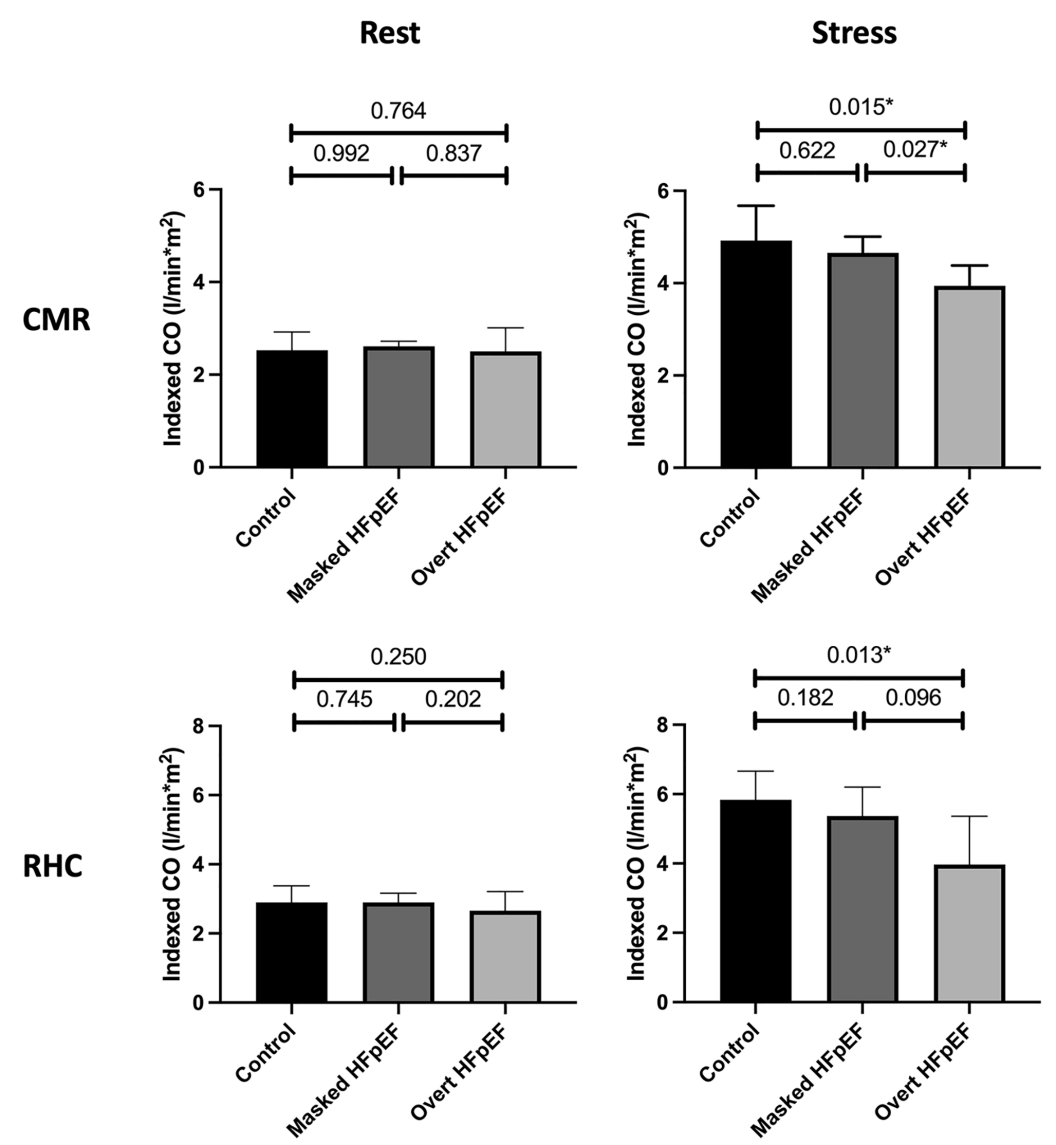
Differences of the indexed cardiac output calculated by real-time CMR and RHC at rest and during exercise stress in patients with masked HFpEF, overt HFpEF and the control group. The top row shows the results of CMR measurements at rest (left) and during exercise stress (right). The bottom row shows the results of RHC measurements at rest (left) and during exercise stress (right). The asterisk indicates statistical significance with a *p*-value < 0.05. Indexed CO – cardiac output indexed to the body surface area, HFpEF - heart failure with preserved ejection fraction, RHC – right heart catheterization, CMR – cardiovascular magnetic resonance imaging

At rest, no differences could be observed between the indexed CO in patients with masked HFpEF, overt HFpEF and the control group (*p* > 0.764). However, during exercise stress, the indexed CO of patients with overt HFpEF was lower compared to the control group (3.9 vs. 4.9 L/min*m2; *p* = 0.015) and compared to patients with masked HFpEF (3.9 vs. 4.7 L/min*m2; *p* = 0.027). There was no difference for the indexed CO between patients with masked HFpEF and the control group (4.7 vs. 4.9 L/min; *p* = 0.622). Measurements during right heart catheterization revealed comparable results, however, during exercise stress patients with overt HFpEF only showed a strong statistical trend to a lower indexed CO compared to patients with masked HFpEF (4.0 vs. 5.4 L/min*m2; *p* = 0.096) (compare Fig. 4).

## Discussion

In heart-failure populations, real-time imaging provides important advantages over conventional CMR regarding patients’ comfort and compliance during the scan [16, 17]. This includes the missing need for breath holds and imaging of patients that cannot completely lay still, for example during exercise stress.

In return, exercise stress testing was shown to be crucial for the diagnosis of HFpEF, as impaired hemodynamics within this patient group might be covered at rest and are only unveiled during exercise stress [18]. While RHC is considered the reference standard for the assessment of exercise-stress haemodynamics [19], real-time CMR is able to provide comparably good diagnostic accuracy in HFpEF patients [9]. Moreover, it has the capabilities to detect subtle cardiac functional differences within this inhomogeneous group of patients and might even aid to identify patients who could be more likely to profit from future therapy approaches [20, 21].

The CO is one of the most important parameters to assess global cardiac function and is being calculated in several clinical conditions [4, 22, 12]. During exercise stress in particular, the CO provides additional use to identify severe conditions in a heart failure populations [23]. Even though real-time CMR yields great premises, its accuracy to measure the CO during exercise stress had yet to be proven.

This study now shows that a CMR-based calculation of the CO using real-time phase-contrast imaging is feasible with excellent reproducibility and good agreement with the reference standard at rest and during exercise stress. Established CMR methods for the calculation of the CO including volumetric stroke volume assessments and conventional phase-contrast imaging showed comparably good agreement with RHC measurements at rest.

In conventional CMR, the calculation of the CO has been implemented already, using conventional phase-contrast imaging or volumetric assessments [7, 22]. We could reconfirm pre-existing literature with good agreement between the mentioned methods and the reference standard of thermodilution.

Despite the good agreement, CO as calculated by the volumetric stroke volume tends to overestimate the CO, as it does not account for retrograde flow like in mitral or aortic regurgitation [24].

In turn, CO as calculated by conventional phase-contrast imaging mostly underestimates the CO compared to the reference standard. This can be explained by the limited temporal resolution of phase-contrast imaging and the nature of the acquisition, where one cardiac cycle is averaged from multiple individual acquisitions and the planning has to be perpendicular to the blood flow [25, 26]. Thereby, peak-flow during systole might be missed during the acquisition and could result in underestimation of CO.

This trend for underestimation was also present for real-time assessments of CO, as real-time phase-contrast imaging suffers from even worse temporal resolution, which compromises stress acquisitions with higher heart rates, in particular [27]. Furthermore, it is important to note that for exercise stress assessments, the mean difference between real-time assessments and RHC increases at a higher CO. This should be considered when evaluating the CO in clinical routine using exercise stress CMR, as the limits of agreement might be slightly different for distinct CO values [28].

Besides the bias towards a lower real-time CO, real-time measurements showed a numerical trend towards a better agreement with the reference standard compared to the other CMR techniques. However, this trend is most likely not significant as confidence intervals of the ICC revealed relevant overlapping.

Except for volumetric LV stroke volume assessments, the heart rate of CMR-based CO calculations at rest and during exercise stress was slightly lower compared to invasive measurements during RHC. The higher heart rate during volumetric assessments could be explained by the repetitive and multiple breath holds required for the acquisition causing stress to the patients. Meanwhile the lower heart rates during conventional and real-time phase-contrast imaging most likely contribute to the underestimation of the CO, as higher heart rates may have resulted in an increased CO, depending on the variability of the stroke volume. For a more technical assessment and validation of the individual techniques, an artificial in-vitro phantom experiment would be required.

Finally, differences between the CO calculated by CMR and RHC could also be partially reason to the time gap between the RHC and CMR examination.

In HFpEF patients, we could further demonstrate the additional benefit of real-time calculations of the indexed CO. Patients with later stages of HFpEF (overt HFpEF) had a lower indexed CO during exercise stress compared to patients with earlier stages of HFpEF (masked HFpEF) and the control group. Importantly, those differences were not present at rest and could be successfully identified using exercise-stress real-time CMR. The same discrimination could be made by right heart catheterization, even though the difference between early and late stages of HFpEF only showed a strong trend without statistical significance. This should be further elaborated in a larger patient cohort.

While the need for invasive measurements of the CO can be dismissed using CMR, it might not necessarily become the standard of care, as the effort for the calculation of a simple parameter does not justify the conduction of a whole CMR scan. However, especially in HFpEF patients, CMR can provide important additional information for diagnosis and prognostication [9, 29, 30, 31] and is being recommended for further evaluation diseases pathogenesis [32]. In these specific clinical indications, real-time rest and exercise stress calculation of the cardiac index can now provide additional reliable, and clinically useful information while avoiding unnecessary invasive procedures at the same time.

### Limitations

This study was a monocentric trial in an experienced core laboratory and might not be reproducible in other centers. Assessment of conventional and real-time phase-contrast imaging can be prone to in-plane motion by breathing or patient movement which potentially corrupts the data unpredictably. Differing heart rates during individual assessments using RHC and CMR might have impacted on the results of CO calculations. Due to the time interval, relevant of the CO due to different pre- or afterload could be possible and potentially impact the results. However, we believe that a time interval of 24 h is sufficient to exclude major variations. Furthermore, RHC and CMR might cause different levels of stress to the patients which could influence the CO during examination.

## Conclusions

The CO at rest and during exercise stress can be assessed using real-time phase-contrast CMR with high reproducibility and good agreement to the invasive reference standard of thermodilution by RHC. Other conventional methods for the calculation of CO using CMR including conventional phase-contrast imaging and the assessment of the volumetric stroke volume showed similar agreement, even though the variability was higher. In multiple conditions, including patients with HFpEF, non-invasive real-time assessments of the CO during exercise can provide clinically useful information, and helps to avoid unnecessary invasive procedures. Next clinical studies need to follow to assess general applicability of the proposed methods.

### Electronic supplementary material

Below is the link to the electronic supplementary material.

Supplementary Material 1

## Data Availability

The datasets used and/or analyzed during the current study are available from the corresponding author on reasonable request.

## Abbreviations

CO: Cardiac output
RHC: Right heart catheterization
CMR: Cardiovascular magnetic resonance imaging
HFpEF: Heart failure with preserved ejection fraction
EF: Ejection fraction
PCWP: Pulmonary capillary wedge pressure
TE: Time of Echo
TR: Time of repetition
ICC: Intra-class correlation coefficient
CoV: Coefficient of Variation
