# Supplementary material for: Assessment of the cardiac output at rest and during exercise stress using real-time cardiovascular magnetic resonance imaging in HFpEF-patients
Source: Int J Cardiovasc Imaging. 2024 Jan 18;40(4):853–62. doi: 10.1007/s10554-024-03054-6 (PMC11052864; doi:10.1007/s10554-024-03054-6)
Supplement: Supplementary file 1 — Supplementary Material 1 [file 10554_2024_3054_MOESM1_ESM.docx]

**Supplemental Data**

Supplemental Table S1: Intra- and inter-observer variability of real-time phase-contrast measurements of the cardiac output.

| **Intra-observer variability** | | | |
| --- | --- | --- | --- |
|  | ICC (95% CI) | P-value | CoV (%) |
| RT-CMR-rest | 0.994 (0.977-0.999) | <0.001 | 2.8% |
| RT-CMR-stress | 0.996 (0.985-0.999) | <0.001 | 2.9% |
| **Inter-observer variability** | | | |
| RT-CMR-rest | 0.998 (0.992-1) | <0.001 | 1.5% |
| RT-CMR-stress | 0.985 (0.940-0.996) | <0.001 | 12.1% |

CMR – cardiovascular magnetic resonance imaging, RT – real-time phase-contrast, ICC – intra-class coefficient, CoV – Coefficient of variation, CI – Confidence interval


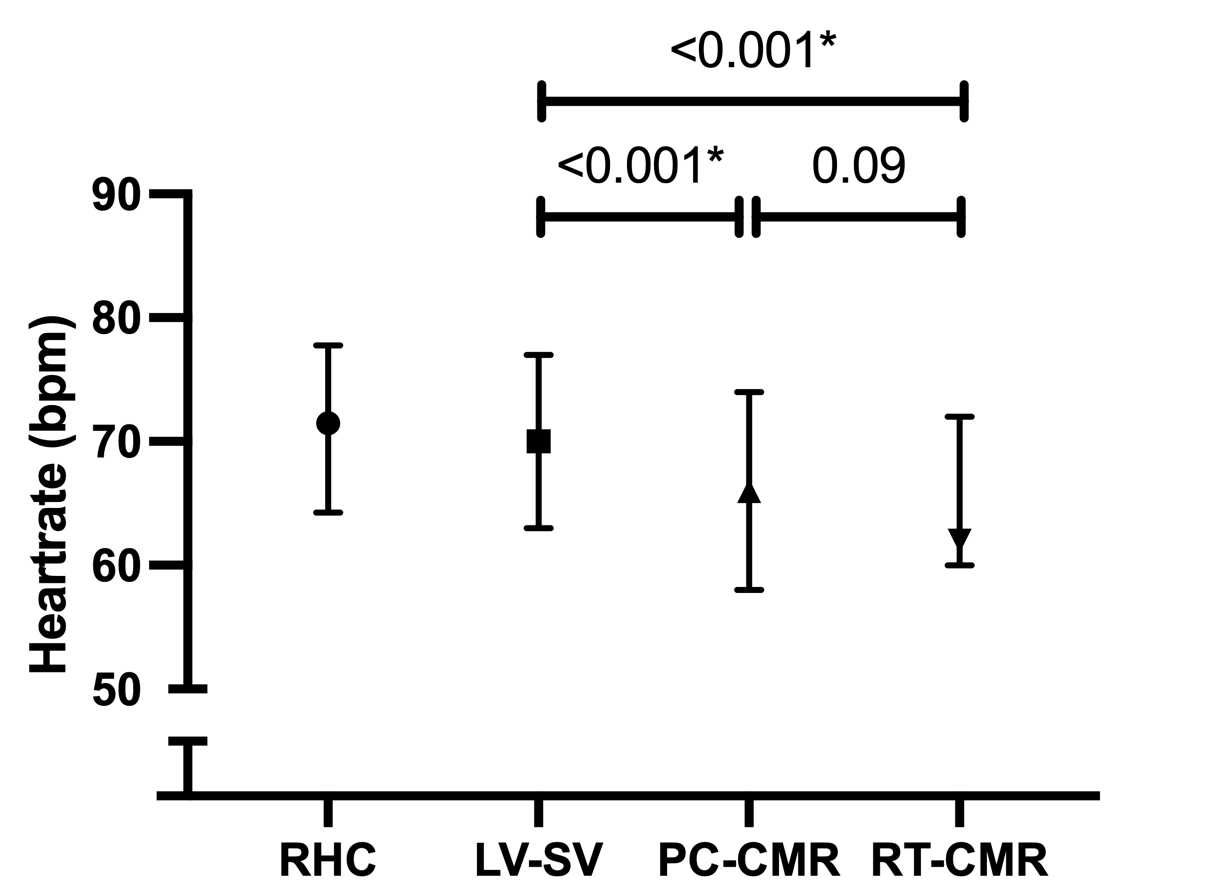


Supplemental Figure 1: Differences of heart rates for the calculation of the cardiac output by the individual methods at rest. Displayed is the median heart rate (± inter quartile range) of the median heart rate for the calculation of the cardiac output at rest by RHC and CMR. P-values below 0.05 are considered statistically significant. RHC – right heart catheterization, LV-SV – left ventricular stroke volume, PC – conventional phase contrast, RT – real-time, CMR – cardiovascular magnetic resonance imaging
